# Pupillometric Evidence for the Decoupling of Attention from Perceptual Input during Offline Thought

**DOI:** 10.1371/journal.pone.0018298

**Published:** 2011-03-25

**Authors:** Jonathan Smallwood, Kevin S. Brown, Christine Tipper, Barry Giesbrecht, Michael S. Franklin, Michael D. Mrazek, Jean M. Carlson, Jonathan W. Schooler

**Affiliations:** 1 Department of Social Neuroscience, Max Planck Institute for Human Cognitive and Brain Sciences, Leipzig, Germany; 2 Department of Physics, University of California Santa Barbara, Santa Barbara, California, United States of America; 3 Department of Psychology, University of California Santa Barbara, Santa Barbara, California, United States of America; University College London, United Kingdom

## Abstract

Accumulating evidence suggests that the brain can efficiently process both external and internal information. The processing of internal information is a distinct “offline” cognitive mode that requires not only spontaneously generated mental activity; it has also been hypothesized to require a decoupling of attention from perception in order to separate competing streams of internal and external information. This process of decoupling is potentially adaptive because it could prevent unimportant external events from disrupting an internal train of thought. Here, we use measurements of pupil diameter (PD) to provide concrete evidence for the role of decoupling during spontaneous cognitive activity. First, during periods conducive to offline thought but not during periods of task focus, PD exhibited spontaneous activity decoupled from task events. Second, periods requiring external task focus were characterized by large task evoked changes in PD; in contrast, encoding failures were preceded by episodes of high spontaneous baseline PD activity. Finally, high spontaneous PD activity also occurred prior to only the slowest 20% of correct responses, suggesting high baseline PD indexes a distinct mode of cognitive functioning. Together, these data are consistent with the decoupling hypothesis, which suggests that the capacity for spontaneous cognitive activity depends upon minimizing disruptions from the external world.

## Introduction

Taking a shower, queuing for coffee, or riding the bus are all everyday tasks with minimal cognitive demands that allow the mind to wander [1], [2]. These common experiences of dual engagement or multi-tasking illustrate that mental activity is not confined to the online processing of sensory information (e.g. thoughts which are more obviously derived from an external referent and are not especially imaginative in nature); it also has an offline mode in which cognition is initiated spontaneously [3]–[5]. This offline mode is imaginative and depends more heavily on the contents of memory than it does on concurrent perceptual information. The fact that the offline mode persists in the face of the distractions of the coffee queue or the bus ride raises a question: Why isn't spontaneous cognitive activity continually disrupted by the information available from perception [6], [7]?

One hypothesis is that the internal train of thought is not interrupted by external events because the mind can reversibly decouple attention from sensory information [3], [8]. This “decoupling” would reduce competition between internally generated representations (offline information) and those derived from perception (online information) [8] by reducing the signal-to-noise ratio of the sensory stream. Critically, decoupling could explain our capacity for orderly, internally guided trains of thought because it would prevent external events from interfering with such offline cognitive processes [8].

Support for the decoupling hypothesis of spontaneous thought comes from evidence that offline thought impairs sensory processing [9], [10] and from the well-established anti-correlation between the so-called default-mode network (DMN) [11] and perceptual or task relevant processes [12]–[14]. Moreover, recent studies that specifically examined episodes of offline thought indicate that both elements of the DMN and aspects of the executive system are simultaneously active. For example, the dorsolateral prefrontal cortex (DLPFC) is recruited in a task during periods of task-unrelated-thought (TUT) [15]. DLPFC is a brain area known to be involved in sustaining cognition in the face of distraction [16]. Similarly, activity in the dorsal anterior cingulate cortex (dACC) has been observed during spontaneous off-task thought [16] and experimenter induced autobiographical planning [17]. While it is plausible that the activation of control processes in periods of decoupled thought indicate that these structures play a role in the control and coordination of offline content [3], this view has been challenged by the suggestion that such activity instead reflects an attempt to reinstate task focus [18].

The present study tests whether the dynamics of pupil diameter (PD) are consistent with the decoupling hypothesis. PD exhibits rapid stimulus-evoked increases following the encoding of external stimuli [19], increases during long term memory retrieval [20], and has been linked to known control processes in the brain (such as DLPFC activity) [21]. These data make PD an ideal covert measure to assess cognitive activity during decoupled thought. In addition, single-cell recordings in primates suggest that changes in PD are correlated with firing rate in the brainstem locus coeruleus (LC) [8], [9], the primary source of brain norepinephrine (NE). This correlation allows the current study to explore the potential role that NE plays in the decoupling of attention from perception; a role for NE in offline cognition seems plausible given the suggestion that it has a mode of operation which facilitates task disengagement [8], [9]. As the precise mechanism by which LC-NE influences PD is unknown, any observed association between PD and cognitive processing should be viewed with caution. Nonetheless, it is increasingly common to use PD as a proximal measure of the LC-NE system [22], [23].

It is important to note that it is currently unknown whether PD (or other indirect measures of cognitive function) will exhibit transient fluctuations that index the experience of specific periods of spontaneous internally guided thought. The current paper, as with other investigations of decoupling [15], assesses the offline mode by comparing baseline activity for classes of events when decoupling is likely to occur to events when it is less likely. We have no way of determining moments of onset for individual episodes of spontaneous thought. If these events occur with no phase relationship to the task structure, trial averaging will destroy their temporal structure and we will not be able to distinguish these events from overall higher baseline activity.

The decoupling hypothesis assumes that the offline and online modes of thought can be understood as different attentional states which result from the competition of internal and external information streams for access to a general purpose, limited capacity attentional workspace [24], [25]. The online mode occurs when external task relevant information forms the focus of attention; as a result internally generated information is prevented access to the workspace. By contrast, during the offline mode, attention to internally generated signals prevents external task relevant information from accessing the workspace. When neuro-cognitive changes occur in response to events in an external task, such changes are assumed to reflect processes relevant to the task (the online mode). On the other hand, neuro-cognitive activity with no obvious trigger in the external environment is assumed to reflect the processing of internally generated signals (the offline mode).

The current paper examines whether PD exhibits these two distinct modes of activity predicted by the decoupling hypothesis: (i) an online mode reflecting *a state of enhanced processing of external task relevant information* in which baseline PD activity is suppressed and transient responses to external events are maximized and (ii) an offline mode involving *a state of enhanced processing of internally generated events* in which transient responses to task events are reduced and baseline levels of PD are enhanced. To examine this hypothesis we formulated five predictions, labeled (P1)–(P5) and summarized in Table 1, on how the dynamics of the online/offline modes of PD should behave.

**Table 1:** Five predictions derived from the decoupling hypothesis of offline thought.

Claim	Prediction	Experiment(s)	Figure(s)
During online cognition attention is coupled to task events	(P1) PD will increase as events in the task are encoded	One, Two	1b, 2a, 4
During offline cognition attention is decoupled from task events	(P2) PD will not increase when events in the task are presented	One, Two	1b, 2a
	(P3) PD will show high baseline activity which is uncoupled from task events	One, Two	1a, 2b
Processing of spontaneously generated mental content requires decoupling of attention from external information	(P4) High baseline PD prior to probes will be indicative of slow correct responses and/or a failure to encode task events	Two, Three	3
States of on/offline cognition are distinct modes of thought	(P5) Baseline PD will show a nonlinear or stepwise relationship to continuous measures of external attention	Two, Three	5

**Figure 1:**
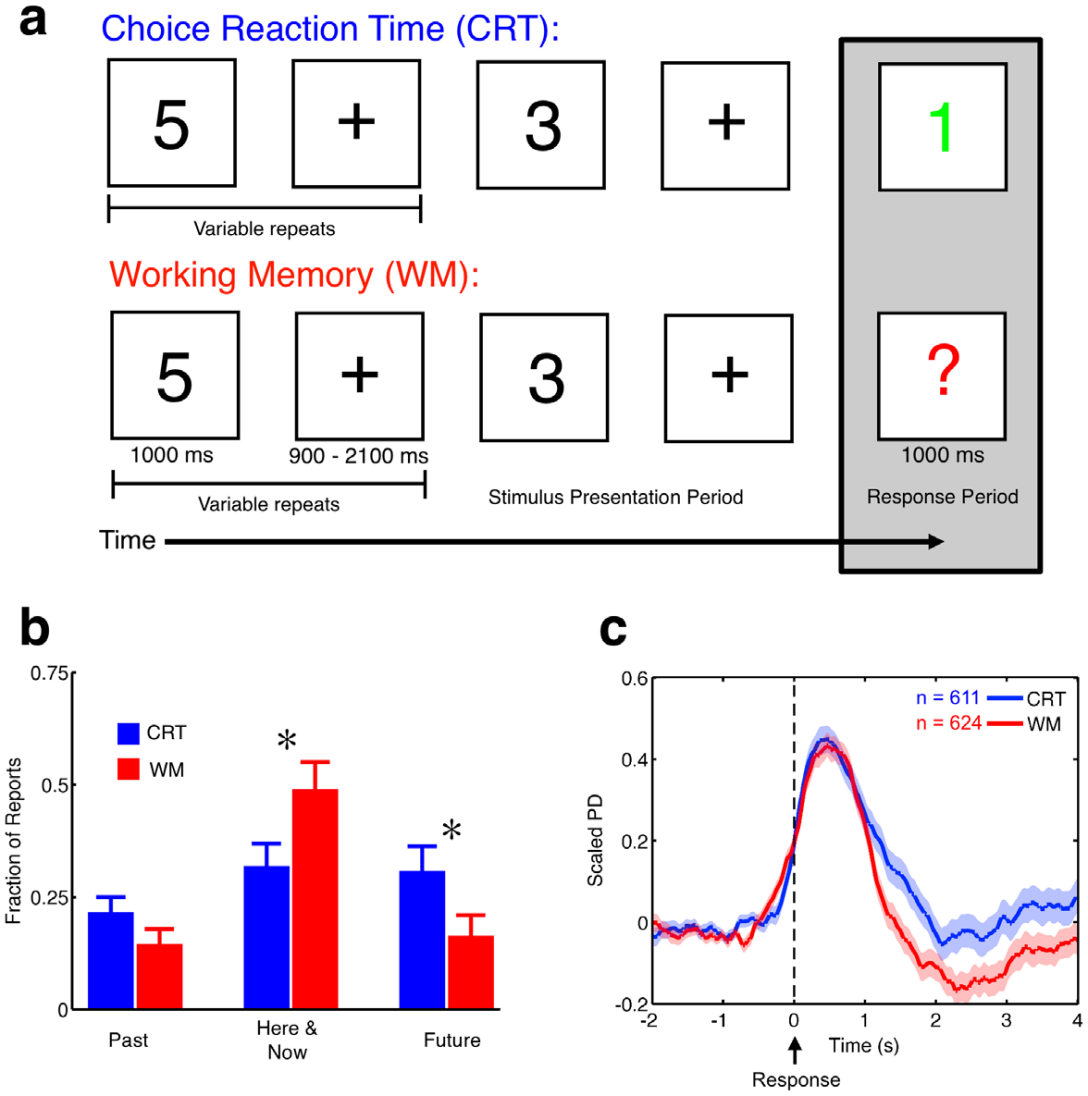
Task description, experience sampling and motor-related pupil response. (**a**) A schematic illustrating the choice reaction time (CRT, blue) and working memory (WM, red) tasks. In the CRT task, correct responses can be made without attention to the non-colored stimuli; this is not true in the WM task. (**b**) Results of Experiment One. Thirty participants who performed above chance for both the WM and CRT tasks were included in the analysis. Participants were asked on 18 occasions whether their attention was focused on the here and now (the task), the future, or the past. A 2×3 analysis of variance (ANOVA) was conducted on the experience sampling data with two factors of task [WM/CRT] and three factors of experience [“Future”/ “Here and Now”/ “Past”]. This analysis indicated a Task×Experience interaction (F (2, 58) = 8.51, p<.001, η^2^ = .23) in which thoughts of the “Here and Now” were more frequent in the WM task (p<0.001) and “Future” thoughts more prevalent in the CRT (p<0.01). “Past” thoughts did not vary across tasks (p = .11). (**c**) Scaled pupil diameter time locked to all responses in the WM and CRT tasks. Thirteen subjects from Experiment Two passed quality control cutoffs. Shaded regions indicate one standard error of the mean, and the response instant at t = 0 is indicated with a dashed line and arrow. In both the WM and CRT tasks the expected robust motor component [49] to pupil size is observed.

To test these five predictions we developed two tasks (Figure 1a) that differed primarily in the mode of cognition required for performance. In the Working Memory (WM) task, participants were presented with a sequence of digits and asked to retain the identity of the most recent number in memory. The participants responded to intermittent probes (a colored “?”) by reporting the parity (odd/even) of the previous number shown. The WM task requires continuous external attention and so satisfactory task performance demands that participants maintain an online external focus. In the Choice Reaction Time (CRT) task, a similar sequence of digits was observed but the intermittent probes only required participants to report the parity of a colored number currently displayed on the screen (see the Materials and Methods section for further details). Hence, no encoding of the non-colored digits was required for high levels of accuracy in the CRT task, and in these periods participants would potentially be able to engage the offline mode with greater frequency than in the WM task. In our experimental paradigm, decoupling could occur in two circumstances: (a) during the processing of the non-colored numbers in the CRT task and (b) during ineffective processing of the same stimuli during the WM task (i.e. slow correct responses or encoding failures). As the “executive failure” view of spontaneous thought [18] could only conceivably be applied to the latter situation [26], common changes in PD in these two circumstances would rule out the interpretation of spontaneous activity as simply returning attention to the task.

## Results

Experiment One used experience sampling [4] to confirm that attention was less task-constrained during performance of the CRT than the WM task (Figure 1b). As hypothesized from previous work [27]–[29], the WM task required that participants maintain focus on the current task environment. In the CRT task participants were comparatively less likely to focus on the present and instead tended to anticipate future events.

**Figure 2:**
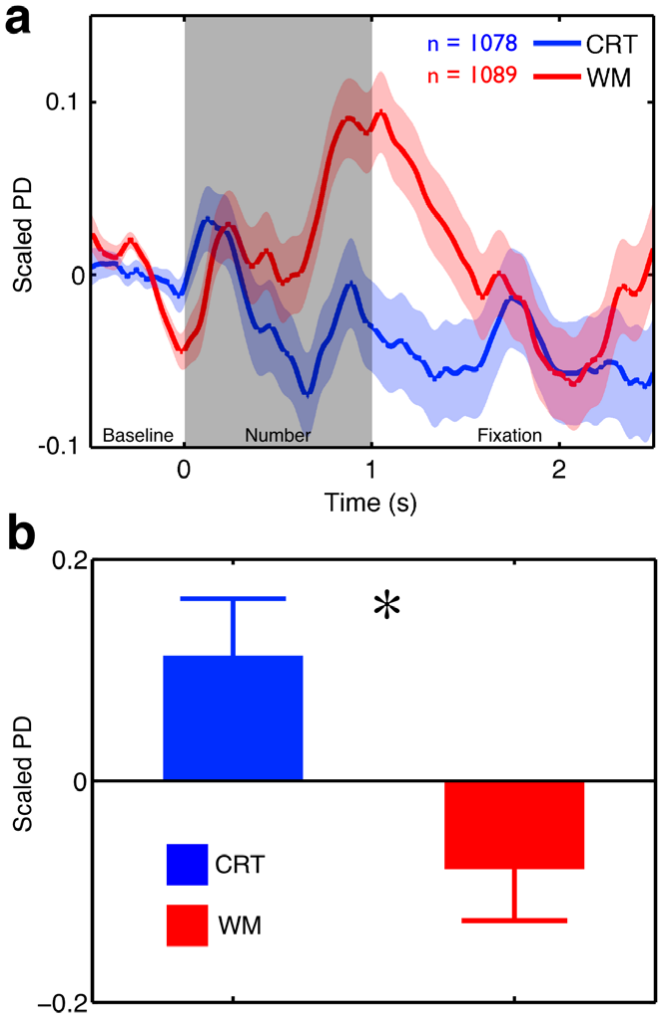
Task differences in baseline and evoked PD in the Working Memory and Choice Reaction Time tasks. (**a**) Thirteen participants from Experiment Two passed the quality control cut-offs and are included in this analysis. Time courses locked to non-probe stimulus presentation were created for each trial for each individual and each task. Values were averaged into ten 250 ms bins and compared using a 2×10 ANOVA with factors of Task [2 levels] and Epoch [10 levels]. A significant Task×Time interaction (F (9, 99) = 3.96, p<.001, η^2^ = .25) indicated differences in the pupil response to non-colored stimuli across the tasks. No other main effects or interactions were statistically significant (all p-values>.05). Contrast analysis examining the difference between conditions indicated the larger evoked response in the WM task accounted for 71% of the variance (F (1, 11) = 28.1, p<.001, η^2^ = .71). (**b**) To examine tonic pupil size we compared the mean non-baselined PD in the 1.5 seconds prior to the presentation of non-colored stimuli for the participants in Experiment Two. An ANOVA including the task order as a comparison revealed that PD was substantially higher in the CRT task than in the WM task (F (1, 11) = 5.48, p<.05, η^2^ = .38). Neither the main effect of task order nor the interaction between task order and task was significant (all p-values>.05).

Experiment Two measured PD for participants performing both tasks to determine (i) if the non-colored stimuli in the WM task would evoke a transient increase in PD (P1) and (ii) if no such increase in PD would be observed in response to these same events in the CRT task (P2). Figure 1c presents the dynamics of PD locked to responses (button presses); the expected increase in PD associated with the motor response is observed in both WM and CRT [49]. Figure 2a presents the dynamics of PD in a 2.5 second epoch after presentation of non-colored stimuli in both tasks. Baseline levels of PD were normalized using the 500 ms interval prior to the non-probe stimulus. A clear evoked response was present in the WM task and absent from the CRT task. Experiment Two therefore confirmed our first two predictions; in the online mode PD shows transient increases coupled to task events (P1) and in the offline mode it does not (P2).

Given that PD activity was uncoupled to the events in the CRT task, we next explored if the same context was accompanied by greater spontaneous cognitive activity (P3). If this were the case, PD should be generally larger during performance of the CRT task than during the WM task (as shown with Experiment One). Figure 2b demonstrates that in the 1.5 s period prior to a non-probe stimulus, average PD in the CRT task was larger than in the WM task.

**Figure 3:**
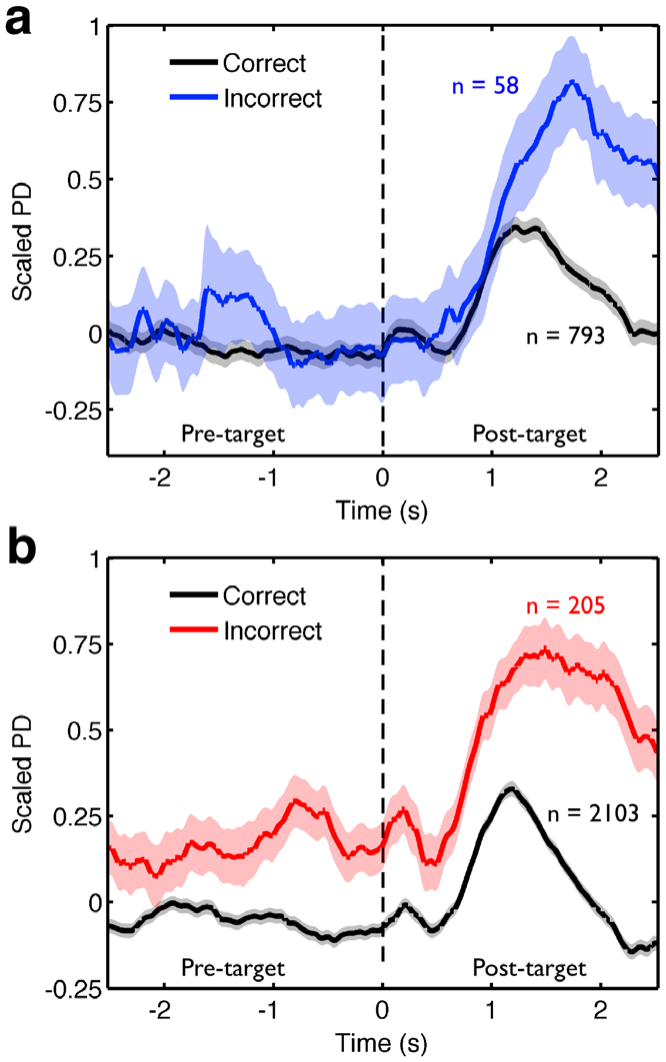
Baseline differences in PD are larger prior to incorrect responses to WM probes. Scaled pupil diameter prior to correct and incorrect responses in the (**a**) CRT and (**b**) WM tasks. Thirteen participants from Experiment Two are included in (**a**) and Twenty-nine participants from Experiments Two and Three in (**b**). Time courses for each trial for each subject, locked to probes, were calculated for correct and incorrect responses for both tasks. (**a**) No pre-target PD differences are evident in the CRT task, and so no additional analysis was performed on those data. (**b**) For the WM task, the 1.5 s interval prior to the probe was divided into ten 150 ms bins. Experiment number (Two/Three) was included as a between participants variable in the ANOVA. A main effect of accuracy (F (1, 25) = 11.0, p<.005, η^2^ = .31) indicated that baseline PD was higher prior to incorrect responses and there was no effect of time, experiment, or their interaction.

Next, if poor external encoding is necessary for spontaneous cognitive activity to persist (P4), high baseline PD levels should be apparent prior to encoding failure during WM responses. PD dynamics in the CRT task were indistinguishable prior to correct and incorrect probes (Figure 3a). However, higher baseline PD prior to incorrect probes was evident in the WM task. To investigate this pre-probe difference with greater power we performed Experiment Three, in which an additional group of participants completed a twenty minute version of the WM task. The data from these subjects were combined with the WM data from the subjects in Experiment Two. Figure 3b shows PD during the 1.5 second window prior to probes binned on subsequent accuracy. Higher baseline PD preceded incorrectly responded WM probes compared to correctly responded probes. Together, Experiments Two and Three show that spontaneous PD activity is accompanied by a reduction in external attention (P4).

**Figure 4:**
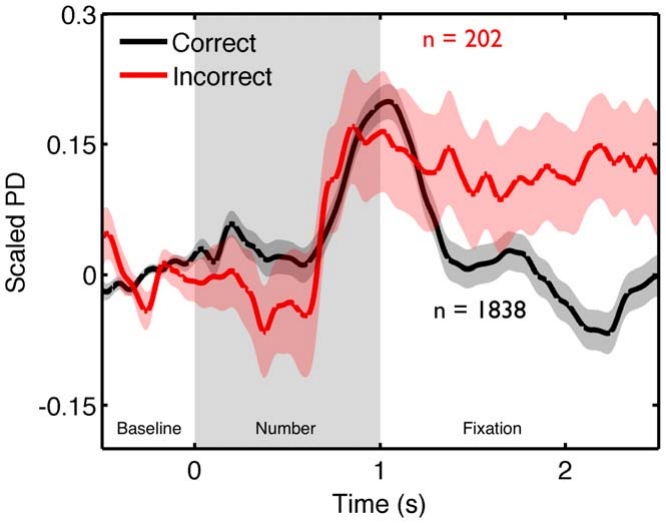
Evoked changes in PD are abnormal during encoding failures in the WM task. Scaled PD prior to correct and incorrect responses in the WM task from Experiments 2 & 3. Twenty six participants passed the quality control cut-offs and made one or more mistakes in the WM task. Three participants were excluded due to PD values greater than 3 SD above the population mean in one or more bins, leaving a total of 23 valid cases. Time courses for each trial for each subject, locked to pre-probe stimuli, were calculated for correct and incorrect responses during the WM task. Values were averaged in ten 250 ms bins and compared using a 2×10 ANOVA with factors of Accuracy [2 levels] and Epoch [10 levels]. Experiment was included as a between participants factor. This analysis yielded the expected Accuracy X Epoch interaction (F (9, 189) = 4.3, p<.01, η^2^ = .17). Contrast analysis indicated that the difference in the PD dynamics between correctly and incorrectly encoded stimuli fitted a cubic pattern that accounted for approximately 24% of the difference in accuracy (F (1, 21) = 7.4, p<.05, η^2^ = .24). This analysis confirms that the difference between situations when external task relevant information was and was not encoded can also be attributed to differences in the evoked pupillary response (P2). No other main effect of accuracy, experiment or their interactions reached statistical significance.

We also investigated whether periods of failed task encoding, leading to subsequent errors, were accompanied by differences in evoked activity in PD (P2). Figure 4 presents the dynamics of PD in a 2.5 s epoch following the last pre-probe stimulus prior to correctly and incorrectly responded probes in the WM task. Baseline levels were normalized using the 500 ms interval prior to the non-probe stimulus. While correct trials were preceded by both a clear dilation and constriction of PD following processing of the target stimulus, incorrect trials showed dilation following stimulus presentation but no subsequent constriction. This analysis confirms that the evoked response to stimuli does change when encoding fails (P2), and in particular there is an absence of PD constriction that accompanies normal encoding.

Finally, we investigated the relationship between baseline PD and degree of task focus. To do so we capitalized on the fact that response time (RT) provides a continuously varying index of the efficiency of external attention. Assuming that offline cognition is associated with decoupling, then large PD should be associated with slower RT (P4). Moreover, based on brain imaging studies suggesting that online and offline thought are discrete modes of cognition [12], we hypothesized (P5) that the relationship between baseline PD and probe RT should reflect this. If the online and offline states of thought are distinct modes of cognitive operation, the relationship between PD and RT should be highly nonlinear, with a sharp rise in PD in a narrow transition zone between two relatively stable states: the online mode – small PD and fast RT – and the offline mode – slow RT and large PD. On the other hand, if PD varies less abruptly with measures of external attention – e.g. linearly increasing with RT – we would question the assumption of distinct online and offline states. In this second case, attention would smoothly switch from online to offline processing, with no clear boundaries between the two states.

**Figure 5:**
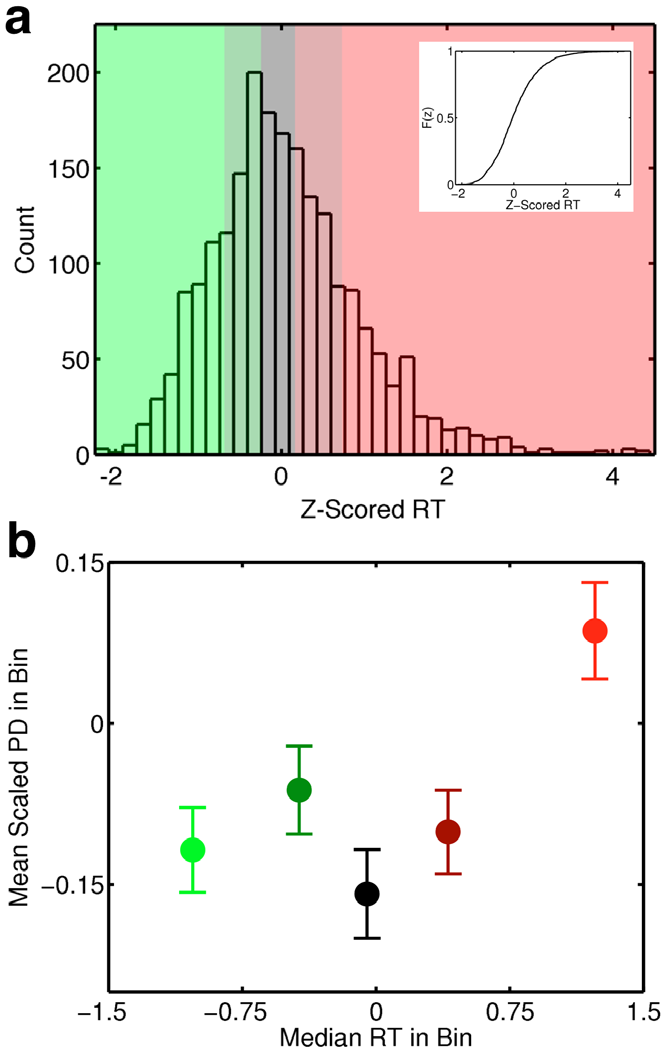
Extremely slow response times to correct WM probes are associated with high baseline PD. (**a**) Reaction times (RTs) to all 2103 correct working memory responses (Experiments Two and Three) were within-subject z-transformed and then pooled. These RTs were divided into five equal mass bins whose boundaries were determined using the cumulative RT distribution (inset) and are denoted by the colored areas in the main panel. (**b**) Binned, mean scaled PD for the 1.5 second window before correct WM probes, plotted against median bin RT. ANOVA including Experiment (Two/Three) as a between-participants variable indicated a significant effect of pupil size on subsequent RT (F (4, 108) = 7.02, p<.001, η^2^ = .19). No additional main effects or interactions were significant (all p-values >.05). Post-hoc comparisons (Bonferroni corrected) among the ANOVA results indicated that the slowest bin differed significantly from all other bins except the fourth bin (all corrected p-values <.05). No other inter-bin differences were significant (all p-values >.05).

Individual subject RTs for correct WM responses in Experiments Two and Three were z-transformed, pooled (Figure 5a, main panel), and divided into five equal bins, with the bin boundaries set using the cumulative RT distribution (Figure 5a, inset). We then computed the mean PD for each bin in the 1.5 second interval prior to the correctly responded probe. Only the very slowest RTs were associated with higher pre-probe baseline PD values; no other RT bins showed significant PD differences. This stepwise or binary relationship suggests that PD activity does index distinct modes of cognition (P5).

## Discussion

Using PD as a neurocognitive marker, we tested five predictions derived from the decoupling hypothesis of offline thought. During online cognition, PD showed phasic increases indicating the processing of task stimuli (P1). In contrast, during periods characterized by offline thought, PD either did not change in response to external stimuli or exhibited abnormal changes (e.g. when encoding failed) (P2) and instead showed high baseline levels of activity decoupled from task events (P3). The same high baseline activity observed in the easier CRT task was also seen prior to task errors and slow responses, both of which indicate reduced attention to perceptual stimuli (P4). Finally, the stepwise relation between RT and PD suggested that online and offline thought represent distinct cognitive modes (P5). Our analysis provides clear support for the decoupling hypothesis of spontaneous thought: PD exhibits a mode of spontaneous activity (i) uncoupled from task events and (ii) associated with differences in the way that external events are processed [3], [6].

While other studies have indicated that offline thought leads to a disengagement from the external world [9], [10], [30]–[33], our data are the first to document that both perceptual coupling and decoupling are apparent in the same neurocognitive measure in a single paradigm. Importantly, the observation of elevated PD in the “non-demanding” CRT task suggests that this activity is involved in a process that facilitates the offline mode, rather than reflecting an attempt to return attention to the task. Instead the elevation in PD during the CRT task is likely to reflect this marker's links to known processes such as memory retrieval [20] and/or forms of affective [34] or social cognition [35], all of which are likely to make up some part of the offline mode [29], [36], [37]. Whether this decoupling represents a specific mechanism which keeps reality separate from mental simulations [8] or arises because of the architecture necessary for conscious thought [24] our data cannot address. However, regardless of the mechanism, decoupling [3], [6] provides an explanation for why the internal train of thought is not continually disrupted; the capacity to disengage cognition from physical reality prevents spontaneously generated mental content from being overshadowed by the continuing stream of sensory information. Without the capacity to decouple attention from perception, conscious thought would always be closely tied to perceptual events and so imaginative acts would be more difficult to engage.

Finally, it is worth speculating about the specific brain systems involved in attentional decoupling. As the DLPFC is (i) recruited during task unrelated thinking [15], (ii) implicated in the maintenance of information in the face of distraction [16], and (iii) associated with increases in PD [21], it is plausible that this brain region is involved in the suppression of irrelevant external information necessary for orderly spontaneous thought to occur. Alternatively, given the close correlation between PD and the dynamics of the brain LC-NE system [8], [9] it is also possible that the LC-NE system plays a role in the decoupling process. The LC-NE system has historically been implicated in maintaining external vigilance [7], [38], [39], recently it has been suggested that it helps agents adaptively balance the need to exploit the opportunities provided by the current environment with those associated with other possible goal opportunities [40], [41]. In the adaptive gain theory (AGT) of NE function [40], [41], the system has three distinct firing modes, closely linked to arousal level, which actively modulate goal pursuit [40], [41]: (i) an “off” state of low LC activity associated with drowsiness and inactivity, (ii) a phasic mode characterized by transient bursts of LC activity synchronized to task events that sustains current goal focus, and (iii) a tonic mode involving high baseline LC activity with a relative absence of task relevant responses that supports goal disengagement. This latter ‘tonic mode’ is typified by high baseline activity and smaller evoked responses and so would be consistent with the empirical characteristics of the “offline” mode presented in the current paper. Further support for the link between the LC-NE system in the process of decoupling comes from the observation that the phasic mode of this system is thought to support both response inhibition and the P3 component of the event-related potential [42] which are characteristics of the online mode [9], [43]–[47]. Despite the appeal of links with either the DLPFC [15] or the LC-NE system [22], [23], PD is a peripheral measure of cognition and further work using more direct brain imaging methods are necessary before the exact neural processes involved in decoupling are known. Nonetheless, given the frequency of occurrence of the offline mode in daily life [1], [2], [48], such future work should consider the specific neuro-cognitive processes that “tune out” the present and so underlie the capacity for the mind to wander.

## Materials and Methods

This study was approved by the University of California, Santa Barbara Psychology Ethics committee under code 09306. Written informed consent was acquired from every participant prior to participation. Pupil size and gaze direction were collected using a Tobii 120 eye tracker (Tobii, Stockholm, Sweden) with a sampling rate of 125 Hz. Participants were seated on a comfortable chair, approximately half a meter from the eye tracker and did not use a chin rest or other immobilization device. Prior to data collection the eye tracker was calibrated to each individual using Tobii Studio. PD was computed for each sample as follows: if measurements from both eyes were recorded as “good” the two pupil diameters were averaged. If only one eye was “good” that measurement was used for PD at that time point. Any remaining times in which both eyes were flagged as “bad” were linearly interpolated. These gaps were generally short (due to either blinks or the hooding of the eye by eyelashes), and we employed a quality control process that rejected subjects with excessive amounts of interpolated data. The data was then median filtered (order 5) in order to remove spikes and low-pass filtered with a cutoff frequency of 10 Hz. Finally, the data were z-transformed within participants; for participants performing both CRT and WM tasks (Experiment Two), both tasks were transformed together to retain task differences.

### Experiment One

Forty-one participants (27 females, Mean Age 18.5(2)) completed the experience sampling study.

### Experiment Two

Twenty-seven healthy (17 females, Mean Age 18.6(3)) participants completed the same versions of both the CRT and WM tasks while being eye tracked.

### Experiment Three

Nineteen participants (Mean Age 19.5(3)) completed a twenty minute version of the WM task while being eye tracked.

No participant in any Experiment had neurological or psychiatric problems, all had normal or corrected to normal vision, and none were color blind. Participants were tested alone in a dim room with stable artificial lighting. Eye tracking participants (Experiments Two and Three) with more than 40% interpolated data were excluded, as were all participants whose accuracy was less than 50%, representing chance in both tasks.

A schematic of the task is shown in Figure 1a. Stimuli were presented against a white background in 40 point Arial font. Non-probe stimuli were presented for 1000 ms followed by a fixation cross which varied in duration from 900–2300 milliseconds (mean duration 1500 ms). Probes (either a “?” during the WM task or a colored number during the CRT task) followed between 2 and 5 black non-probe stimuli, and were presented in color (red or green, counterbalanced across tasks and participants) to reduce perceptual demands of probe detection. Probes were equally likely to follow an even or an odd digit in both tasks. Participants were instructed to respond only to the colored events, and to use the mouse to indicate if the number was odd (press the left button) or even (press the right button); no responses were made to the non-probes in either task. Task duration for all experiments was twenty minutes; each ten minute section contained 48 probes. Task order was counterbalanced in Experiments One and Two.

In the experience sampling study (Experiment One) all features of the tasks were identical with the exception that 18 odd/even probes were replaced with experience sampling probes. These probes asked participants to indicate whether, in the period immediately prior to the probe, they were thinking about (i) the task/here and now, (ii) task unrelated personal events in the past, (iii) task unrelated personal events in the future, or (iv) abstract task unrelated thoughts with no temporal focus. Responses were recorded using the computer keyboard.
